# Bronchoalveolar Lavage Fluid Protein Expression in Acute Respiratory Distress Syndrome Provides Insights into Pathways Activated in Subjects with Different Outcomes

**DOI:** 10.1038/s41598-017-07791-8

**Published:** 2017-08-07

**Authors:** Maneesh Bhargava, Kevin Viken, Qi Wang, Pratik Jagtap, Peter Bitterman, David Ingbar, Chris Wendt

**Affiliations:** 1 Division of Pulmonary, Allergy, Critical Care and Sleep Medicine, University of Minnesota Medical School, Minneapolis, USA; 2 Biostatistical Design and Analysis Center, Clinical and Translational Science Institute, University of Minnesota, Minneapolis, USA; 3 Biochemistry, Molecular Biology and Biophysics, University of Minnesota Medical School, Minneapolis, USA; 4 Minneapolis VAMC, Minneapolis, USA

## Abstract

Acute respiratory distress syndrome (ARDS) is associated with high mortality. We sought to identify biological pathways in ARDS that differentiate survivors from non-survivors. We studied bronchoalveolar lavage fluid (BALF) from 36 patients with ARDS (20 survivors, 16 non-survivors). Each sample, obtained within seven days of ARDS onset, was depleted of high abundance proteins and labeled for iTRAQ LC-MS/MS separately. Protein identification and relative quantification was performed employing a target-decoy strategy. A variance weighted *t*-test was used to identify differential expression. Ingenuity Pathway Analysis was used to determine the canonical pathways that differentiated survivors from non-survivors. We identified 1115 high confidence proteins in the BALF out of which 142 were differentially expressed between survivors and non-survivors. These proteins mapped to multiple pathways distinguishing survivors from non-survivors, including several implicated in lung injury and repair such as coagulation/thrombosis, acute phase response signaling and complement activation. We also identified proteins assigned to fibrosis and ones involved in detoxification of lipid peroxide-mediated oxidative stress to be different in survivors and non-survivors. These results support our previous findings demonstrating early differences in the BALF protein expression in ARDS survivors vs. non-survivors, including proteins that counter oxidative stress and canonical pathways associated with fibrosis.

## Introduction

Acute respiratory distress syndrome (ARDS) occurs as a response to infectious or inflammatory triggers and is characterized by acute tachypnea, refractory hypoxia, and loss of lung compliance^1^. Although a variety of conditions are triggers for ARDS, common risk factors include pneumonia (59%), extrapulmonary sepsis (16%), and aspiration (14.2%)^2^. Despite widespread adoption of lung protective ventilation^3^, early use of muscle relaxants^4^, extracorporeal membrane oxygenation^5^ and prone ventilation^6^, case fatality rates remain at 30–40%^2, 7–10^.

Regardless of the cause of ARDS, there is an unmet need to develop tools to reliably assess the mortality risk of patients early in ARDS evolution in order to design interventions to improve survival rates. The current definition of ARDS is based on physiological derangement^11^ and does not identify causal mechanisms. However, there is heterogeneity in disease susceptibility^12–16^ and manifestations. Only 50% of cases classified as ARDS have diffuse alveolar damage on biopsy^17^. Other lung-specific responses differ with respect to the degree of hypoxia^2^, ventilator driving pressure^18^, lung stiffness^17^, and collagen deposition^19–21^. A greater understanding of the differences in the disease biology that accounts for the variability in ARDS manifestations is critical to improve outcomes for ARDS patients^22^. Sophisticated modeling using latent class analysis of the ARDS Network cohort has identified subphenotypes in ARDS^23^. The subphenotypes with higher plasma concentrations of inflammatory markers are associated with an increased prevalence of sepsis requiring vasopressors and an inadequate response to therapeutic interventions such as fluid restrictive therapy^24^. Understanding differences in the underlying biologic processes that influence survival of ARDS patients early in the course of the illness could promote precision-medicine and exploration of new or existing therapeutic agents to improve survival^22^.

We have previously reported early differences in bronchoalveolar lavage fluid (BALF) between ARDS survivors and non-survivors^25^, although the study was conducted on pooled BALF and with only a single mass-spectrometry (MS) experiment. Here, we sought to identify biological processes and canonical pathways that differ in ARDS survivors and non-survivors by characterizing individual BALF samples using state-of-the-art label-based semi-quantitative protein expression profiling using iTRAQ (isobaric tagging for relative and absolute quantification) labeling in combination with fixed-gene set enrichment analysis. This study represents a critical first step in our effort to identify proteomic molecular endotypes in ARDS and identify potential signaling pathway targets to reduce ARDS-related mortality.

## Results

### Characteristics of Study Subjects

In this study, we characterized ARDS BALF samples from 20 subjects who survived and 16 subjects that died during their hospital stay. Five patients (3 survivors and 2 non-survivors) had indirect lung injury from either sepsis (n = 4) or pancreatitis (n = 1), whereas 31 cases had direct lung injury such as pneumonia or aspiration pneumonia (17 survivors and 14 non-survivors). Table 1 outlines the risk factors for ARDS and previous pulmonary history for the study subjects. The median time to death after bronchoscopy in the non-survivors was seven days (IQR = 16.5 days). The median time from ARDS onset (defined as the day of intubation) to bronchoscopy was 2 days. The length of intensive care unit (ICU) stay was not significantly different between the two groups; although the length of hospital stay was lower in non-survivors, this did not reach statistical significance (Table 2).

**Table 1:** ***Pulmonary History and Clinical Risk Factors for ARDS in Study Subjects.***

	ARDS Survivors	ARDS Non-Survivors
**ARDS Risk Factor**		
Direct lung injury		
Aspiration pneumonia	3	1
Pneumonia		
Gram positive	3	1
Gram negative	2	2
Mixed gram positive & negative	2	0
Polymicrobial (fungal & bacterial)	1	1
Unknown (no cultures)	4	4
Viral	2	2
Fungal	0	3
Indirect lung injury		
Pancreatitis	1	0
Sepsis	2	2
**Past Pulmonary History**		
Fungal lung infection	1	0
Lung transplant	2	2
COPD	1	0
Reactive airways disease	3	0
Pulmonary hypertension	1	0
ILD	0	2
Sarcoidosis	0	1
Pleural disease (effusion/pneumothorax)	0	2
Lung cancer	0	1

COPD, Chronic Obstructive Pulmonary Disease; ILD Interstitial Lung Disease.

**Table 2:** ***Demographic and Clinical Characteristics of Study Subjects.***

	ARDS Survivors	ARDS Non-survivors	p-value^A^
Number	20	16	
Age (years)	42 (32–57)	59 (45–72)	0.01
Sex (Male/Female), n	12/8	7/9	0.33
ICU length of stay	16 (12–26)	15 (10–26)	0.86
Hospital length of stay	29 (18–39)	21 (11–37)	0.28
ARDS Day	2.0 (1.0–4.5)	2.0 (1.0–8.0)	0.76
PF ratio	95 (72–154)	76 (61–168)	0.65
BAL Leukocytes (/μl)	332 (216–753)	280 (160–801)	0.68
BAL Neutrophills (%)	66 (46–80)	40 (27–83)	0.50
BAL Lymphocytes (%)	2.5 (0–5.3)	0.5 (0–3.8)	0.28

Values are median (inter-quartile range), unless otherwise noted.

^A^P-values are from the Mann-Whitney U test for comparing medians, except for sex which uses a Chi-square test on the percentage of male/female.

BAL, Bronchoalveolar lavage; ICU, Intensive Care Unit; PF Ratio, PO_2_: FiO_2_ ratio.

ARDS day: the day BALF was obtained relative to ARDS onset as defined by mechanical ventilation initiation.

Survivors were younger than non-survivors (mean age 42 vs. 59 years, p = 0.01), but there were no statistically significant differences between the two groups in the time from ARDS onset to BALF collection (ARDS day) or in BALF leukocyte, neutrophil or lymphocyte counts (Table 2). The global internal standard consisted of BALF from 27 subjects who had respiratory failure from various etiologies, including pleural disease, mediastinal mass, myopathy/polyneuropathy, interstitial lung disease without exacerbation, radiation pneumonitis, infectious pneumonia, congestive heart failure and ARDS (one patient).

### Proteins Identified by Database Search

The Protein-Pilot Proteomics System Performance Evaluation Pipeline Software (PSPEP) false discovery rate (FDR) summary showing the number of spectra, peptides and the proteins identified at 1% global FDR for the six iTRAQ experiments is shown in Table 3. Six iTRAQ liquid chromatography tandem MS (LC-MS/MS) runs identified 850, 606, 1055, 865, 976, and 879 proteins per run, respectively (Supplemental Table S1). The database search using the combined RAW files from all six LC-MS/MS experiments resulted in the identification of 1189 proteins at a local FDR ≤ 5% (Supplemental Table S2, master protein list tab). After manually removing misidentified proteins, reverse matches, contaminants, and proteins that were not completely removed by the high abundance protein depletion column, we retained 1115 unique proteins for further analysis. The fold change for these proteins can be found in Supplemental Table S2.

**Table 3:** ***PSPEP Protein Summary Report for Proteins Identified at ≤1% Global False Discovery Rate.***

	Spectra	Peptides	Proteins
iTRAQ LC-MS/MS 1	37651	11623	850
iTRAQ LC-MS/MS 2	21183	7761	606
iTRAQ LC-MS/MS 3	25849	11865	1055
iTRAQ LC-MS/MS 4	24577	9061	865
iTRAQ LC-MS/MS 5	26623	11037	976
iTRAQ LC-MS/MS 6	26111	10389	879
Combined iTRAQ LC-MS/MS 1 to 6	160073	21148	1284

LC, liquid chromatography; MS, mass spectrometry.

### Differentially Expressed Proteins between ARDS Survivors and Non-survivors

For each iTRAQ eightplex, the fold changes for proteins identified were available with low (113–117) and high (118–121) reporter ion channels as the reference. Thus, we performed this analysis using the lower reporter ion channel and the higher reporter ion channel as a reference to calculate the relative abundance for all six iTRAQ LC-MS/MS experiments. Controlling for a q-value of ≤ 0.05 and using two internal standard reporter ion channels, we identified 181 and 166 proteins to be differentially expressed when we used the low or high reporter ion channel as the reference for calculation of fold change, respectively (Supplemental Table S3), with 142 proteins common between the two comparisons (Supplemental Table S3).

Proteins with the highest differential expression that were more abundant in survivors included galectin-10 (Q05315, log fold = −0.21 ± 0.12 vs 2.20 ± 0.17), cystatin-SA (P09228, log fold = 0.04 ± 0.02 vs 2.45 ± 0.26), glutamine gamma-glutamyltransferase 2 (P21980, log fold = 0.28 ± 0.09 vs 1.82 ± 0.24), prosaposin (C9JIZ6, log fold = 0.01 ± 0.07 vs 1.51 ± 0.21), and cathepsin D (P07339, log fold = 0.50 ± 0.11 vs 1.99 ± 0.19). Proteins with the highest differential expression with higher abundance in non-survivors included alpha-amylase 1 (P04745, log-fold 1.14 ± 0.20 vs −0.74± 0.04), club-cell secretory protein (P11684, log fold 2.03 ± 0.11 vs. 0.43 ±0.11), BPI fold-containing family B member 2 (Q8N4F0, log fold = 1.20 ± 0.19 vs 0.31 ± 0.09), metallothionein-2 (P02795, log fold = 2.31±vs 0.87± 0.15), and myoglobin (P02144, log fold 1.15 ±I 0.14 vs 0.13± 0.10).

Among the proteins that were more abundant in survivors were different isoforms of aldehyde dehydrogenase, alcohol dehydrogenase, and aldo-keto reductases (Table 4). Specifically, we observed significantly higher levels of isoform 2 of alcohol dehydrogenase 2, aldo-keto reductase family 1 C1, glutathione peroxidase 3, aldehyde dehydrogenase mitochondrial. There also was a statistical trend towards a higher abundance of glutathione-s-transferase A2 (q-value = 0.08).

**Table 4:** ***Proteins of Interest that Demonstrate a Difference in Expression between Survivors and Non-Survivors in Low Control.***

Uniprot Accession Number	Protein Name	Non-survivors		Survivors		p-value
		N	Weighted log fold change (mean ± SE)	N	Weighted log fold change (mean ± SE)	
Metabolic enzymes						
P40394-2	Isoform 2 of Alcohol dehydrogenase class 4 mu/sigma chain	13	0.26 ± 0.09	17	1.74 ± 0.17	6.36E-06
Q04828	Aldo-keto reductase family 1 member C1	13	0.15 ± 0.08	17	0.93 ± 0.12	3.54E-04
P22352	Glutathione peroxidase 3	16	0.43 ± 0.08	20	1.15 ± 0.16	3.96E-03
P05091	Aldehyde dehydrogenase, mitochondrial	6	0.37 ± 0.04	6	1.19 ± 0.21	3.19E-02
P09210	Glutathione-*S*-trasferase A2	15	0.70 ± 0.15	15	1.21 ± 0.18	0.08
Proteins mapped to fibrosis pathways						
P20908	Collagen alpha-1 (V) chain	12	0.39 ± 0.08	12	1.19 ± 0.16	4.04E-03
P22692	Insulin-like growth factor binding protein 4	16	1.12 ± 0.14	20	0.52 ± 0.06	7.17E-03
P19320	Vascular cell adhesion protein 1	13	0.58 ± 0.13	17	0.16 ± 0.05	2.73E-02
P02751	Fibronectin	16	0.00 ± 0.07	20	0.30 ± 0.09	3.73E-02
H7BXV5	Collagen alpha-1 (XVIII) chain	13	0.82 ± 0.12	17	0.37 ± 0.07	1.81E-02
Proteins with differential expression that also were identified in our previous study						
P11684	Club cell secretory protein (uteroglobin)	16	2.03 ± 0.11	20	0.43 ± 0.11	1.68E-09
P00450	Ceruloplasmin	16	0.17 ± 0.10	20	0.60 ± 0.09	1.90E-02
P00747	Plasminogen	16	0.24 ± 0.14	20	0.68 ± 0.14	6.77E-02
P15311|	Ezrin	16	0.63 ± 0.12	20	0.32 ± 0.06	6.24E-02
P06702	S100-A9	16	0.36 ± 0.13	20	0.76 ± 0.13	8.01E-02
P01008	Antithrombin-III	16	0.12 ± 0.12	20	0.35 ± 0.11	2.04E-01
P00748	Coagulation factor XII	16	0.19 ± 0.10	20	0.34 ± 0.08	2.66E-01

### Biological Relevance of the Differentially Expressed Proteins

**Figure 1:**
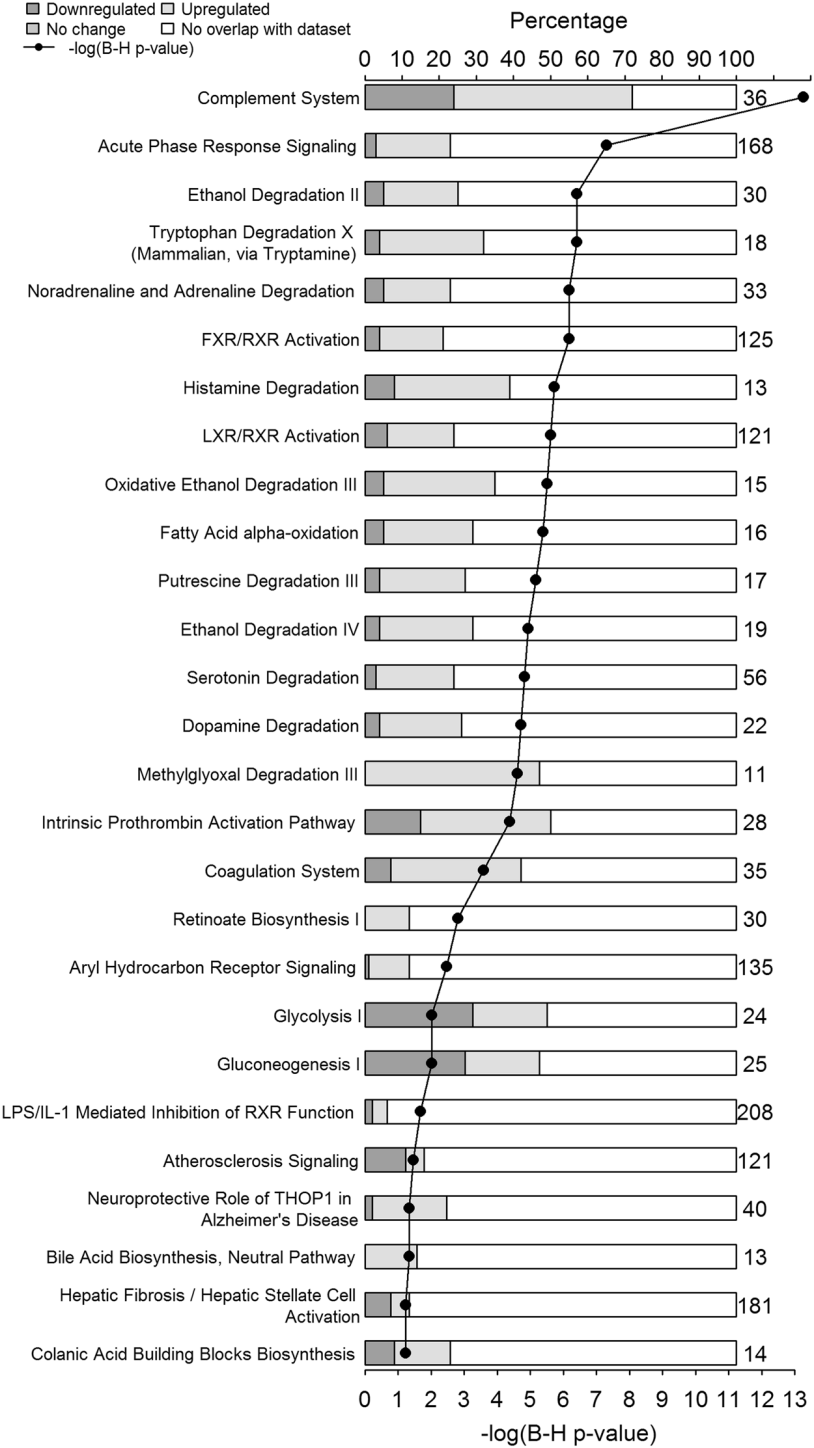
**Canonical Pathways represented by proteins differentially expressed between ARDS survivors and non-survivors**. Ingenuity Pathway Analysis (IPA) canonical pathways most significantly changed in ARDS survivors compared to non-survivors. The stacked bar chart displays the percentage of proteins in the canonical pathway that are more (light gray), less (dark gray) abundant in survivors or absent (white) our dataset. The secondary x-axis shows the −log of Benajmni-Hochberg corrected p-value indicating the statistical significance of each over-represented pathway (cutoff >1.3) and the line with solid diamonds is the −log of corrected p-value for that pathway. The numerical value on right side of each stacked bar is the number of genes in that canonical pathway in the IPA knowledgebase.

We performed IPA core analysis to identify the canonical pathways which map to the differentially expressed proteins that met a threshold q-value of ≤0.05. The pathways that met the statistical threshold described in the methods (−log [B-H p-value] ≥ 1.3) and the proteins assigned to each canonical pathway are listed in Table 5. Figure 1 shows the degree of enrichment and the number of proteins with increased or decreased abundance for each pathway in survivors compared with non-survivors. The z-score was available for Complement System (1.667), Acute Phase Response Signaling (2.238), LXR/RXR Activation (3.162), Intrinsic Prothrombin Activation Pathway (1.00) and Coagulation System (0.447). Of the pathways listed in Table 5, only pathways that participate in fibrosis (Hepatic Fibrosis/Hepatic Stellate Cell Activation) were represented in an independent core analysis done on proteins that were more abundant in non-survivors than in survivors. Certain pathways such as Complement Activation, Fatty acid alpha oxidation, Histamine Degradation, Oxidative Ethanol degradation were mapped by separate IPA core analysis by the proteins that were high in survivors and also by some proteins that were high in non-survivors (although with limited coverage of the pathway). The remaining canonical pathways were mapped by proteins more abundant in survivors.

**Table 5:** ***IPA Canonical Pathways Represented by Proteins Differentially Expressed Between Survivors and Non-Survivors.***

Ingenuity Canonical Pathways	−log (B-H p-value)^A^	Gene Symbols	Protein Names
Complement System	1.34E01	C4A/C4B, CR1, CD55, MBL2, C1S, C9, MASP1, C8B, C6, C1QB, CFH, C5	Complement C4-A/C4-B, Complement receptor type 1, complement decay-accelerating factor (CD antigen CD 55), Mannose-binding protein C, Complement C1s subcomponent, Complement component C9, Mannan-binding lectin serine protease 1, Complement component C8 beta, Complement component C6, Complement C1q subcomponent subunit B, Complement factor H, Complement C5
Acute Phase Response Signaling	7.41E00	ITIH3, FN1, C1S, C9, CP, SERPINA3, C5, SERPIND1, C4A/C4B, KLKB1, MBL2, ITIH4, CRP, AGT	Inter-alpha-trypsin inhibitor heavy chain H3, Fibronectin, Complement C1s subcomponent, Complement component C9, Ceruoplasmin, alpha-1-antichymotrypsin, Complement C5, Heparin cofactor 2, Complement C4-A/C4-B, Plasma kallikrein, Mannose-binding protein C, Inter-alpha = trypsin inhibitor heavy chain H4, C-reactive protein, Angiotensinogen
Ethanol Degradation II	6.41E00	ALDH2, AKR1A1, ADH7, ALDH1A1, ALDH3B1, ALDH3A1, ALDH9A1	Aldehyde dehydrogenase mitochondrial, Aldo-keto reductase family 1 member A1, Alcohol dehydrogenase class 4 mu/sigma, Retinal dehydrogenase 1, Aldehyde dehydrogenase family 3 member B1, Aldehyde dehydrogenase dimeric NADP-preferring, 4-trimethylaminobutyraldehyde dehydrogenase
Tryptophan Degradation X (Mammalian, via Tryptamine)	6.41E00	ALDH2, AKR1A1, ALDH1A1, ALDH3B1, ALDH3A1, ALDH9A1	Aldehyde dehydrogenase mitochondrial, Aldo-keto reductase family 1 member A1, Retinal dehydrogenase 1, Aldehyde dehydrogenase family 3 member B1, Aldehyde dehydrogenase dimeric NADP-preferring, 4-trimethylaminobutyraldehyde dehydrogenase
Noradrenaline and Adrenaline Degradation	6.24E00	ALDH2, AKR1A1, ADH7, ALDH1A1, ALDH3B1, ALDH3A1, ALDH9A1	Aldehyde dehydrogenase mitochondrial, Aldo-keto reductase family 1 member A1, Alcohol dehydrogenase class 4 mu/sigma, Retinal dehydrogenase 1, Aldehyde dehydrogenase family 3 member B1, Aldehyde dehydrogenase dimeric NADP-preferring, 4-trimethylaminobutyraldehyde dehydrogenase
FXR/RXR Activation	6.24E00	C4A/C4B, KNG1, L CAT, ITIH4, C9, FBP1, PLTP, GC, A1BG, CLU, AGT	Complement C4-A/C4-B, Kininogen-1,, Lecithin-cholesterol acyltransferase, Inter-alpha = trypsin inhibitor heavy chain H4, Complement component C9, Fructose-1 6-bisphosphatase 1, Phospholipid transfer protein, Vitamin D-binding protein, Alpha-1B-glycoprotin, Clusterin, Angiotensinogen
Histamine Degradation	5.74E00	ALDH2, ALDH1A1, ALDH3B1, ALDH3A1, ALDH9A1	Aldehyde dehydrogenase mitochondrial, Retinal dehydrogenase 1, Aldehyde dehydrogenase family 3 member B1, Aldehyde dehydrogenase dimeric NADP-preferring, 4-trimethylaminobutyraldehyde dehydrogenase
LXR/RXR Activation	5.49E00	C4A/C4B, KNG1, LCAT, ITIH4, C9, PLTP, GC, A1BG, CLU, AGT	Complement C4-A/C4-B, Kininogen-1, Lecithin-cholesterol acyltransferase, Inter-alpha = trypsin inhibitor heavy chain H4, Complement component C9, Fructose-1 6-bisphosphatase 1, Phospholipid transfer protein, Vitamin D-binding protein, Alpha-1B-glycoprotin, Clusterin, Angiotensinogen
Oxidative Ethanol Degradation III	5.49E00	ALDH2, ALDH1A1, ALDH3B1, ALDH3A1, ALDH9A1	Aldehyde dehydrogenase mitochondrial, Retinal dehydrogenase 1, Aldehyde dehydrogenase family 3 member B1, Aldehyde dehydrogenase dimeric NADP-preferring, 4-trimethylaminobutyraldehyde dehydrogenase
Fatty Acid α-oxidation	5.38E00	ALDH2, ALDH1A1, ALDH3B1, ALDH3A1, ALDH9A1	Aldehyde dehydrogenase mitochondrial, Retinal dehydrogenase 1, Aldehyde dehydrogenase family 3 member B1, Aldehyde dehydrogenase dimeric NADP-preferring, 4-trimethylaminobutyraldehyde dehydrogenase
Putrescine Degradation III	5.27E00	ALDH2, ALDH1A1, ALDH3B1, ALDH3A1, ALDH9A1	Aldehyde dehydrogenase mitochondrial, Retinal dehydrogenase 1, Aldehyde dehydrogenase family 3 member B1, Aldehyde dehydrogenase dimeric NADP-preferring, 4-trimethylaminobutyraldehyde dehydrogenase
Ethanol Degradation IV	5.04E00	ALDH2, ALDH1A1, ALDH3B1, ALDH3A1, ALDH9A1	Aldehyde dehydrogenase mitochondrial, Retinal dehydrogenase 1, Aldehyde dehydrogenase family 3 member B1, Aldehyde dehydrogenase dimeric NADP-preferring, 4-trimethylaminobutyraldehyde dehydrogenase
Serotonin Degradation	5E00	ALDH2, AKR1A1, ADH7, ALDH1A1, ALDH3B1, ALDH3A1, ALDH9A1	Aldehyde dehydrogenase mitochondrial, Aldo-keto reductase family 1 member A1, Alcohol dehydrogenase class 4 mu/sigma, Retinal dehydrogenase 1, Aldehyde dehydrogenase family 3 member B1, Aldehyde dehydrogenase dimeric NADP-preferring, 4-trimethylaminobutyraldehyde dehydrogenase
Dopamine Degradation	4.76E00	ALDH2, ALDH1A1, ALDH3B1, ALDH3A1, ALDH9A1	Aldehyde dehydrogenase mitochondrial, Retinal dehydrogenase 1, Aldehyde dehydrogenase family 3 member B1, Aldehyde dehydrogenase dimeric NADP-preferring, 4-trimethylaminobutyraldehyde dehydrogenase
Methylglyoxal Degradation III	4.61E00	AKR1A1, AKR1C1/AKR1C2, AKR1C3, AKR1B10	Aldo-keto reductase family 1 member A1, C1/C2, C3, and B10
Intrinsic Prothrombin Activation Pathway	4.27E00	KNG1, KLKB1, F9, F5, COL18A1	Kininogen-1, Plasma kallikrein, Coagulation factor IX, V, Collagen alpha-1(XVIII)
Coagulation System	3.8E00	KNG1, KLKB1, F9, F5, SERPIND1	Kininogen-1, Plasma kallikrein, Coagulation factor IX, V, Heparin cofactor 2
Retinoate Biosynthesis I	2.83E00	ADH7, ALDH1A1, AKR1C3, AKR1B10	Alcohol dehydrogenase class 4 mu/sigma, Retinal dehydrogenase 1, Aldo-keto reductase family 1 member C3, B10
Aryl Hydrocarbon Receptor Signaling	2.65E00	TGM2, CTSD, ALDH1A1, NQO1, ALDH3B1, ALDH3A1, ALDH9A1	Protein-glutamine gamma-glutamyltransferase 2, Cathespin D, Retinal dehydrogenase 1, NAD(P)H dehydrogenase [quinone] 1, Aldehyde dehydrogenase family 3 member B1, A1, 4-trimethylaminobutyraldehyde dehydrogenase
Glycolysis I	1.94E00	FBP1, GAPDH, ALDOC	Fructose-1 6-bisphosphatase 1, Glyceraldehyde-3-phosphate dehydrogenase, Fructose-bisphosphate aldolase C
Gluconeogenesis I	1.91E00	FBP1, GAPDH, ALDOC	Fructose-1 6-bisphosphatase 1, Glyceraldehyde-3-phosphate dehydrogenase, Fructose-bisphosphate aldolase C
LPS/IL-1 Mediated Inhibition of RXR Function	1.62E00	ALDH1A1, FABP4, ALDH3B1, FABP5, PLTP, ALDH3A1, ALDH9A1	Retinal dehydrogenase 1, Fatty acid-binding protein adipocyte, Aldehyde dehydrogenase family 3 member B1, Fatty acid-binding protein epidermal, Phospholipid transfer protein, Aldehyde dehydrogenase dimeric NADP-preferring, 4-trimethylaminobutyraldehyde dehydrogenase
Atherosclerosis Signaling	1.41E00	VCAM1, L CAT, COL18A1, CLU, PRDX6	Vascular cell adhesion protein 1, Lecithin-cholesterol acyltransferase, Collagen alpha-1(XVIII), Clusterin, Peroxiredoxin-6
Neuroprotective Role of THOP1 in Alzheimer’s Disease	1.37E00	KNG1, SERPINA3, AGT	Kininogen-1, Alpha-1-antichymotrypsin, Angiotensinogen
Bile Acid Biosynthesis, Neutral Pathway	1.37E00	AKR1C1/AKR1C2, AKR1C3	Aldo-keto reductase family 1 member A1, C1/C2, C3
Hepatic Fibrosis/Hepatic Stellate Cell Activation	1.36E00	COL5A1, IGFBP4, VCAM1, FN1, COL18A1, AGT	Collagen alpha1-(V), Insulin-like growth factor binding protein 4, Vascular cell adhesion protein 1, Fibronectin, Collagen alpha-1(XVIII), Angiotensinogen
Colanic Acid Building Blocks Biosynthesis	1.34E00	TSTA3, UGP2	GDP-L-fucose synthase, UTP—glucose-1-phosphate uridylytransferase

^A^Threshold for significance is -log (B-H) p-value ≥ 1.3, which is equivalent to a corrected p-value ≤ 0.05.

We also identified proteins with differential expression that are assigned to pathways that participate in fibrosis (Table 4) such as collagen alpha-1(V), insulin-like growth factor binding protein 4, vascular cell adhesion protein 1, fibronectin, and collagen alpha-1(XVIII). Additionally, there were several other proteins that participate in the pathophysiology of fibrosis, although they were not differentially expressed in survivors versus non-survivors. These included monocyte differentiation antigen CD14, angiotensinogen, insulin-like growth factor II, insulin-like growth factor binding protein 3, metalloproteinase inhibitor 1 and 2, 72 kDa type IV collagenase, matrix metalloproteinase-9, and collagens alpha-1(I), alpha-2(I), alpha-1(III), alpha-1(V), and alpha-3 (VI).

### Proximity Extension Assay for Proteins Participating in Inflammation

As a validation of our iTRAQ findings, we selected five proteins identified in the iTRAQ experiments that were also present in the Olink PEA panel. These proteins were growth-regulated alpha protein (P09341-CXCLI), macrophage colony-stimulating factor 1 (P09603), monocyte chemotactic protein 1 (P13500), Protein S100-A12 (P80511), and Interleukin-18 (Q14116). The Olink results were consistent with those of the MS experiments in finding no difference in the protein level between survivors and non-survivors for growth-regulated alpha protein or S100 A-12. Additionally, the Olink analysis found no statistically significant difference in macrophage colony-stimulating factor 1 or monocyte chemotactic protein 1 by study group. This comparison was not possible with MS because high-quality quantitative information was not available; monocyte chemotactic protein 1 was only present in one of the six iTRAQ experiments, and error factor was not available for macrophage colony-stimulating factor 1 in two of the three iTRAQ MS/MS experiments. By MS, Interleukin-18 was identified in the BALF of only 12 ARDS subjects (6 survivors and 6 non-survivors), and although the levels were higher in non-survivors, the difference did not achieve statistical significance (q-value = 0.22). However, with Olink, Interleukin-18 was identified in all ARDS cases studied except one non-survivor and higher levels were seen in non-survivor compared to survivors (5.8 ± 0.88 vs. 4.9 ± 1.1, p = 0.04).

## Discussion

In this study, we successfully applied label based high-resolution protein expression profiling tools to comprehensively characterize BALF from individual cases of ARDS and identified differences between survivors and non-survivors. We also confirmed the finding of a coordinated response in survivors and a fibrotic signature in non-survivors. PEA results provide analyte level validation for a subset of inflammatory proteins that are present in ARDS BALF.

Oxidative stress occurs in ARDS due to the underlying pathophysiology, but is exacerbated by mechanical ventilation given the high oxygen fractions in inspired gas that is required to maintain adequate oxygenation. In many critically ill patients, limiting oxidative stress by either restricting oxygen exposure or treating with supplemental antioxidants is beneficial and can improve mortality^26, 27^. However, in ARDS the use of antioxidants has produced mixed results. Some studies show improved gas exchange^28^, shorter duration of ventilation^28, 29^, and improved APACHE II score^30^ while others show no benefit^31, 32^. In our prior work, Gene Ontology (GO) enrichment analysis, a form of over-representation analysis of fixed-gene sets, identified proteins more abundant in survivors that mapped to cellular cation homeostasis and iron ion homeostasis^25^ and some of these proteins are antioxidant and cytoprotective proteins regulated by nuclear factor, erythroid 2 like (NRF2)-antioxidant response elements (ARE)^33^. In the current study, the IPA core analysis revealed differential expression of several metabolic canonical pathways, such as fatty acid alpha-oxidation and ethanol degradation, represented by many of the same proteins (Table 4), including several that are thought to be enzymes involved in the detoxification of lipid peroxidation derived aldehydes^34^ and are regulated by NRF-ARE. These proteins include aldehyde dehydrogenase, alcohol dehydrogenase, aldo-keto reductases, and glutathione-*S*-transferase, which were found to be more abundant in survivors. Lipid aldehydes such as Malondialdehyde (MDA), hydroxynonenal (HNE), and acrolein are formed in conditions of oxidative stress^35^ by oxidation of polyunsaturated fatty acids. These byproducts are highly reactive, forming adducts with DNA or proteins^35–37^ to impact cellular homoeostasis by inactivating critical enzymes such as Na,K-ATPase^38–41^. The damage due to protein modification can be mitigated by mechanisms that convert the lipid aldehydes into less reactive alcohols by aldo-keto reductases or alcohol dehydrogenases, or to acids by aldehyde dehydrogenase. These reactions can occur spontaneously (phase 1 metabolism) or can be catalyzed by glutathione-*S*-transferase (phase 2 metabolism). In this study, enzymes that participate in detoxification of the lipid peroxides are higher in survivors suggesting a higher capacity to counteract lipid aldehydes. The fact that proteins that participate in phase 1 and 2 metabolisms of toxic byproducts of peroxidation are more abundant in survivors suggests that the host response to mitigate oxidative stress governs outcomes in ARDS. The precise lipid aldehyde and the specific mechanism will need further characterization for developing specific treatments in the ARDS cases lacking mechanisms to limit oxidant stress.

Similar to our previous findings in which differentially expressed proteins mapped to the GO term ‘collagen metabolic processes’^25^, several of the differentially expressed proteins identified in the current analysis mapped to pathways that participate in fibrosis. Although these proteins map to ‘hepatic fibrosis’, this is likely due to a better annotation of these proteins in liver fibrosis, and the fibrotic signature in our study represents a fibrotic response in the lung. In a recent autopsy study, pulmonary fibrosis was present in 51% of ARDS cases of pulmonary origin but only in 20% of cases of extrapulmonary origin^17^; collagen content in the lung is higher in pulmonary than in extrapulmonary ARDS^17, 42^. A majority of the cases in our study had direct pulmonary injury as a trigger which could explain why many of our subjects demonstrated a difference in matrix proteins. In conditions associated with pulmonary fibrosis, the extracellular matrix (ECM) and alveolar microenvironment regulate profibrotic genes^43–46^. Our previous study observed several ECM proteins, including collagen type 1, III and V, mucin 5a, and matrix metalloproteinase 9, to have a higher abundance in non-survivors^25^. In the current study, we identified proteins that participate in the pathophysiology of fibrosis, including ECM proteins such as collagens alpha-1 (XVIII), alpha-1 (I), alpha-2 (I), alpha-1 (III), alpha-1 (V), alpha-3 (VI), and fibronectin 1. Several of these proteins are involved in transforming growth factor beta and insulin-like growth factor-mediated pulmonary fibrosis. Of these, angiotensinogen, collagen alpha-1 (XVIII), collagen alpha-1 (V), fibronectin 1, insulin-like growth factor binding protein 4, and vascular cell adhesion protein 1 had differential expression between survivors and non-survivors. Though speculative, our findings regarding differential protein expression suggest a difference in host response mediated by transforming growth factor beta or insulin-like growth factor leading to early collagen deposition in non-survivors. The exact significance of this finding will need further evaluation. It is thus possible that mediators of fibrosis that increase after disease onset^47^ could be detected much earlier in BALF than with histology on lung tissue.

Congruent with the findings from our previous study, the variance weighted fold change showed higher levels of ceruloplasmin, plasminogen, antithrombin III and coagulation factor XII in survivors, whereas the level of club cell secretory protein (uteroglobin) was higher in non-survivors (Table 4). In contrast to our prior study with pooled BALF, we did not find higher moesin levels in non-survivors in this study but did observe that ezrin (a member of the ezrin, radixin and moesin family of proteins) was high in non-survivors. We acknowledge that the levels of some proteins that met the statistical threshold in our prior study – such as thioredoxin and S100-A9 – did not achieve statistical significance in this study; in fact, the direction of change was different between the two studies. It is possible that differences in the respective study populations or the inherent variability in protein levels over the course of illness could account for these differences. Utilizing a uniform time for sample collection from prospectively enrolled subjects would be beneficial in future studies. Despite the observed differences from our previous study mentioned above, the pathways and biologic processes represented by differentially expressed proteins between ARDS survivors and non-survivors found in the current study are consistent with those seen in the previous study with pooled patients^25^.

The results of this study should be viewed in light of several limitations. First, the outcome of interest is binary (survival versus non-survival) and can be influenced by some factors not controlled for in our study, such as comorbid conditions. In addition, although there was sufficient BALF to perform MS on individual samples, the overall sample size was relatively limited. Further, the MS method used data-dependent acquisition (DDA) which is biased against low-abundance protein quantification. Future work would benefit from utilizing data-independent acquisition (DIA) such as Sequential Windowed Acquisition of all Theoretical Fragment Ion Mass Spectrometry (SWATH-MS). Also, given the relative heterogeneity between survivors and non-survivors, there may be potential confounders which could have influenced the observed results. Despite these limitations, the current study provides new hypotheses for testing in a larger study cohort. A larger sample size would also permit the use of bootstrapping and other cross-validation tools for modelling phenotypic differences in heterogeneous and complex diseases such as ARDS.

Results from the current study validate several proteins previously reported to be upregulated in survivors in pooled BALF studies^25^. These proteins include plasminogen, antithrombin III, coagulation factor XII, and ceruloplasmin. Although all demonstrated an increase in expression, not all were statistically significant in the current analysis, likely due to the limited sample size in this study. One protein, club-cell secretory protein, was significantly higher among non-survivors, as was observed in our previous study.

This study furthers our knowledge about the differences in the biological processes activated in ARDS survivors and non-survivors. It also identifies potential future research areas, including determining the role of lipid peroxides and lung fibrosis. Given that a variety of conditions can lead to ARDS, optimal treatment of patients may differ by the mechanism active in individual subjects^48^, thus supporting phenotyping of ARDS for personalizing care^23, 49^.

## Methods

### Study Population

Eligible subjects consisted of individuals with ARDS as defined by American-European Consensus Conference (AECC) criteria^50^ treated at the University of Minnesota Medical Center between May 2009 and September 2012. This study included 36 subjects who underwent a clinically indicated bronchoscopy within seven days of their ARDS diagnosis and for whom excess BALF was available. The study was designed before the publication of the criteria established by the ARDS Definition Task Force (Berlin definition)^11^. Pooled BALF from 27 subjects who participated in our previous study^51^ was used as the control to determine the relative protein abundance and as global internal standards for comparison across the different iTRAQ LC-MS/MS experiments. A standard protocol for bronchoscopy was used for BALF collection^25, 51^.

ARDS subjects were divided into two groups: those who survived until hospital discharge (survivors) and those who died prior to discharge (non-survivors). Subjects with a history of HIV or viral hepatitis were excluded from the study.

### Sample processing for protein profiling

After collection, all BALF samples were immediately placed on ice and centrifuged at 500 g at 4 °C for 10 minutes within 60 minutes of collection. Cell and debris free supernatant were stored at −80 °C and did not undergo any thaw-freeze cycles until sample processing.

We employed label-based semi-quantitative proteomics using eightplex iTRAQ reagent^52^ for our study. iTRAQ multiplexed sets of reagents for quantitative protein analysis place isobaric mass labels at the N-termini and lysine side chains of peptides. All resulting peptides are isobaric and chromatographically indistinguishable, but yield signature of reporter ions following collision induced dissociation that can be used to identify and quantify peptides in a digest mixture.

To compare protein abundance across different LC-MS/MS experiments and as a reference for determining relative protein abundance, we used the global internal standard. In each LC-MS/MS experiment, two iTRAQ reporter ion channels contained the global internal standard. The remaining six channels contained individual study samples (i.e., we performed 6 separate iTRAQ LC-MS/MS experiments to characterize the 36 ARDS cases in the study). The labeling strategy for the 36 BALF samples studied is outlined in Supplemental Table S4. To prevent reporter ion signal (channel) bias, survivor and non-survivor samples were randomly placed in different iTRAQ reporter ion channels in each experiment.

BALF containing at least 8 mg of protein (Bradford reagent, Bio-Rad cat#500-0006) was processed separately employing a protocol previously published with minor modifications^25, 51^. BALF was concentrated and desalted using Amicon 3-MWCO filters (Millipore Ireland Ltd, Cork, Ireland), depleted of high abundance proteins (Seppro IgY 14 spin column, Sigma-Aldrich, cat # SEP010) with appropriate buffer exchanges for trypsin digestion and labeling with iTRAQ followed by two-dimensional (2D) LC-MS/MS. The adequacy of the trypsin digestion was confirmed by analysis of 3 µg of the digested peptides with linear trap quadrupole MS (LTQ-MS). Equal amounts of the remaining peptide mixtures within each experiment were labeled with eightplex iTRAQ reagent per the manufacturer’s (AB Sciex, Framingham, MA) instructions^51^. 2D LC-MS/MS of the iTRAQ-labeled peptides was conducted as previously described^51^. Data-dependent MS acquisition was performed on a Thermo Scientific LTQ Orbitrap Velos system with higher energy collision induced dissociation (HCD) activation for peptide tandem MS. LC and MS experimental details were the same as previously reported^51^.

### Database search for protein identification and quantification

RAW files generated directly from the mass spectrometer were imported into Galaxy-P platform^53^ for protein identification and quantification^25, 51^. Galaxy-P has also been used for proteogenomics analysis^54–56^ and metaproteomics studies^57, 58^. The MGF files were searched against the target-decoy version of Human UniProt database along with the contaminant sequences from the **c**ommon **R**epository of **A**dventitious **P**roteins (cRAP, http://www.thegpm.org/crap/) (88,304 sequences in total; Date Aug 1, 2014) using ProteinPilot version 4.5. PSPEP-FDR reports and protein and peptide-level summaries were generated within Galaxy-P as previously described^54–58^. The MS proteomics data have been deposited to the ProteomeXchange Consortium^59^ via the PRIDE partner repository with the dataset identifier PXD002672.

In each eightplex iTRAQ LC-MS/MS experiment, we used the global internal standard as a reference to determine the relative abundance and labeled two iTRAQ reporter ion channels in order to provide guidance regarding the FDR of quantitative differences (within each experiment). Specifically, the relative abundance of all proteins identified in each experiment when compared using the two reporter ion channels labeled with the global internal standard should be equal to 1; proteins that show a statistically significant difference thus indicate false positives. In our six iTRAQ LC-MS/MS experiments, the number of proteins that had a statistically significant difference and therefore false positive were 8, 15, 53, 11, 25 and 8, respectively, suggesting an adequate quantitative assessment.

The results of multiple iTRAQ LC-MS/MS experiments were aligned to compare protein-levels using Protein Alignment Template vs. 2.00p (AB Sciex)^51, 60^. For this alignment, we created a ‘reference master list’ by performing a database search using RAW files from all six iTRAQ LC-MS/MS experiments. To ensure that the proteins in this list were of high ID quality, a *local* FDR ≤5% was used as a threshold for protein identification in the reference master list as per the recommendation of the Protein Alignment Template. For the creation of feature tables with quantitative values, the threshold of ≤5% *global* FDR was used for individual sets for the six-iTRAQ LC-MS/MS experiments. Protein Alignment Template resulted in aligning the ratios (fold change), p-values and error factors for the proteins across replicate experiments by using accession numbers of isoforms within the protein summary and UniProt database. However, these included misidentified proteins (e.g., bovine albumin, pig trypsin, bovine casein), matches to reverse (decoy) protein sequences^61^, the contaminant protein sequences from the **c**ommon **R**epository of **A**dventitious **P**roteins, and the proteins that were not completely removed by the IgY 14 depletion column or their protein fractions such as immunoglobulin chains. These proteins were manually removed from the final protein list (Supplemental Table S2, removed proteins tab).

To further investigate the difference in the BALF inflammatory proteins in ARDS survivors, we performed Olink proseek proximity extension assay (PEA)^62^. The inflammatory panel of the Olink PEA was performed on 29 un-depleted BALF samples (non-survivors = 14, survivors = 15). BALF was concentrated and desalted using a 3KD MW Amicon centrifugal filter (Millipore, Cork, IRL). Bradford assay (Bio-Rad, USA) determined the protein concentration of samples diluted with Dulbecco’s PBS to 0.5ug/uL and an equal amount (0.5 ug) of proteins per the manufacturer’s instructions.

### Statistics

Identification of differentially expressed proteins between ARDS survivors and non-survivors involved several steps that were similar to our prior studies with minor modifications^51^. We controlled for multiple comparisons by FDR corrected q-value ≤ 0.05. Log-transformed fold changes for all proteins that were identified in at least two-iTRAQ LC-MS/MS experiments for which error factors were available were compared between ARDS survivors and non-survivors using weighted two-sample t-tests with weights being the inverse variance of the log-transformed fold changes. The Storey method^63^ was used to control the FDR. This analysis was carried out using the lower reporter ion channel (113–117) and the higher reporter ion channel (117–121) in Statistical Analysis Software (version 9.3, SAS Institute Inc., Cary, NC).

To gain insight into the biological significance of differentially expressed proteins, we performed functional analysis using Ingenuity Pathway Analysis (IPA® QIAGEN, Redwood City www.quiagen.com/ingenuty Build 321510 M, Version 21249400). This analysis was performed on proteins with a q-value of ≤ 0.05 as the cutoff for differential expression. IPA core analysis was performed using the difference of the weighted log fold change between survivors and non-survivors. Thus, the proteins that were higher in survivors had a positive value and the proteins that were lower had a negative value. We focused on canonical pathways that met a Benjamini and Hochberg (B–H)–corrected p-value (obtained using the right-tailed Fisher exact test) of ≤0.05 (equivalent to −log [B–H p-value] ≥ 1.3). Additionally, IPA uses the z-score algorithm that informs the activation states of canonical pathways with ability to predict activation, inhibition, no change or inability to predict the activation state^64^. In our study, a positive score predicts activation in ARDS survivors. Several of the pathways in our dataset did not have a z-score assigned to them to predict their activation state.

### Study Approval

The University of Minnesota Institutional Review Board (IRB) Human Subjects Committee approved this study (IRB # 0812M5623) and all methods were performed according to the relevant guidelines. Because we utilized excess BALF from clinically indicated bronchoscopies in this study, and as there was minimal risk to study participants, the IRB approved a waiver of consent for this study.

## Electronic supplementary material

Supplemental Table S1

Supplemental Table S2

Supplemental Table S3

Supplemental Table S4
