# Supplementary material for: Bronchoalveolar Lavage Fluid Protein Expression in Acute Respiratory Distress Syndrome Provides Insights into Pathways Activated in Subjects with Different Outcomes
Source: Sci Rep. 2017 Aug 7;7:7464. doi: 10.1038/s41598-017-07791-8 (PMC5547130; doi:10.1038/s41598-017-07791-8)
Supplement: Supplementary file 4 — Supplemental Table S4 [file 41598_2017_7791_MOESM4_ESM.doc]

**Bronchoalveolar Lavage Fluid Protein Expression in Acute Respiratory Distress Syndrome Provides Insights into Pathways Activated in Subjects with Different Outcomes**

Maneesh Bhargava, Kevin Viken, Qi Wang, Pratik Jagtap, Peter Bitterman, David Ingbar, Chris Wendt

| **Supplemental Table 4: Labeling strategy for the iTRAQ experiments** | | | | | | | | |
| --- | --- | --- | --- | --- | --- | --- | --- | --- |
|  | Reporter ion channel 113 | Reporter ion channel 114 | Reporter ion channel 115 | Reporter ion channel 116 | Reporter ion channel 117 | Reporter ion channel 118 | Reporter ion channel 119 | Reporter ion channel 121 |
| iTRAQ-1 | Control | 6851  Survivor 1 | 6960  Survivor 2 | Control | 7331  Survivor 3 | 6869  Non-survivor 1 | 6944  Non-survivor 2 | 7648  Non-survivor 3 |
| iTRAQ-2 | 7530  Survivor 4 | Control | 7857  Survivor 5 | 8021  Survivor 6 | Control | 7738  Non- survivor 4 | 7875  Non-survivor 5 | 8073  Non-survivor 6 |
| iTRAQ-3 | 8127  Survivor 7 | 6853  Survivor 8 | Control | 6857  Survivor 9 | 7573  Non- survivor 7 | Control | 8097  Non- survivor 8 | 8030  Non- survivor 9 |
| iTRAQ-4 | 6959  Survivor 10 | 7084  Survivor 11 | 7106  Survivor 12 | Control | 8254  Non-Survivor 10 | 7665  Non- survivor 11 | Control | 7033  Non- survivor 12 |
| iTRAQ-5 | 7834  Survivor 13 | 7971  Survivor 14 | 8129  Survivor 15 | 6860  Non- survivor 13 | Control | 7329  Non- survivor 14 | 8219  Non- survivor 15 | Control |
| iTRAQ-6 | Control | 6980  Survivor 16 | 7912  Survivor 17 | Control | 6981  Survivor 18 | 7548  Survivor 19 | 7157  Non- survivor 16 | 6863  Survivor 20 |
